# Research on Red/Near-Infrared Fluorescent Carbon Dots Based on Different Carbon Sources and Solvents: Fluorescence Mechanism and Biological Applications

**DOI:** 10.3390/nano15020081

**Published:** 2025-01-07

**Authors:** Jun Song, Minghao Kang, Shujian Ji, Shuai Ye, Jiaqing Guo

**Affiliations:** 1 State Key Laboratory of Radio Frequency Heterogeneous Integration, College of Physics and Optoelectronic Engineering, Key Laboratory of Optoelectronic Devices and Systems of Ministry of Education and Guangdong Province, Shenzhen University, Shenzhen 518060, China; songjun@szu.edu.cn (J.S.); kk1512863660@163.com (M.K.); jsj13724358050@163.com (S.J.); 2 Medical Engineering and Technology College, Xinjiang Medical University, Urumqi 830011, China

**Keywords:** R/NIR-CDs, biological applications, fluorescence mechanisms, synthesis, optical properties

## Abstract

Fluorescent carbon dots, especially red/near-infrared-emitting CDs, are becoming increasingly important in the field of biomedicine. This article reviews the synthesis, fluorescence mechanisms, and biological applications of R/NIR-CDs, emphasizing the importance of carbon source and solvent selection in controlling their optical properties. The formation process of CDs is classified, and the fluorescence mechanisms of CDs are summarized, involving carbon core states, surface states, molecular states, and cross-linking enhanced emission effects. This article also highlights the applications of R/NIR-CDs in bioimaging, biosensing, phototherapy, and drug delivery. The final section discusses challenges and prospects.

## 1. Introduction

Carbon dots (CDs) are a class of carbon-based fluorescent nanomaterials with particle sizes smaller than 10 nm, exhibiting photoluminescent properties. They were initially discovered in 2004 by Xu et al. during the purification process of oxidized single-walled carbon nanotubes [1]. Over the past two decades, the field of CDs has undergone rapid development. Initially, the fluorescence quantum yield (QY) of CDs was only about 10% [2]. However, there have been significant improvements in optical properties, including QY [3]. Furthermore, CDs have demonstrated broad application prospects, such as multicolor luminescence [4,5,6,7,8], ion detection [9,10,11], light-emitting diodes [12,13,14,15], photocatalysis [16,17], and more.

In addition to the optical properties of CDs, their excellent biocompatibility and low toxicity have also attracted researchers’ attention [18]. Researchers are not only studying how to adjust the absorption/emission window of CDs, but also actively applying them in various biotechnological applications, such as bioimaging [19,20,21,22,23], sensing [24,25,26,27], drug delivery [19,28,29,30,31], antimicrobial agents [32,33,34,35], anticancer agents [36,37,38], and so on.

Compared to the ultraviolet/visible light range, the red/near-infrared (R/NIR) light range (650–1800 nm) holds greater advantages in the biomedical field [39]. This is partly due to the spontaneous fluorescence of biological tissues mainly being blue or green. Probes emitting in the red/near-infrared light range can help mitigate autofluorescence interference in biological imaging, thereby effectively improving the signal-to-noise ratio [40]. Additionally, the lower scattering of R/NIR light within biological tissues allows for greater tissue penetration depth, making it well-suited for in vivo deep imaging and therapy. However, most CDs currently emit wavelengths smaller than 600 nm, with absorption bands within the ultraviolet range, making it challenging to achieve red/near-infrared biological applications. The use of such CDs may lead to strong tissue absorption, shallow tissue penetration, high autofluorescence interference, and severe photodamage to tissues and cells [18]. Therefore, the development of R/NIR-CDs is crucial for the further advancement of CDs in the biomedical field. In 2015, Ge et al. prepared R/NIR-CDs using polythiophene propionic acid (PPA) as a precursor [41]. Their study demonstrated for the first time that R/NIR-CDs could serve simultaneously as fluorescence, photoacoustic, and thermal therapeutic tools for cancer diagnosis and treatment in live mice. In 2016, Pan et al. first prepared R/NIR-emitting CDs that could be excited by a NIR femtosecond laser (850 nm), with an emission peak at 683 nm [42]. Their excellent water dispersibility, narrow emission band, and high QY make them highly promising in the field of biomedical imaging.

In the current scientific research context, the synthesis mechanism of R/NIR-CDs has not been fully elucidated [43]. Therefore, there is an urgent need for a more rigorous and comprehensive analysis of existing experiments. Among the numerous methods for preparing near-infrared CDs, hydrothermal/solvothermal synthesis and microwave-assisted synthesis stand out as mainstream preparation technologies due to their significant advantages such as rich precursor material selection, easy regulation of carbon dot properties, simple equipment requirements, and high yields.

**Figure 1:**
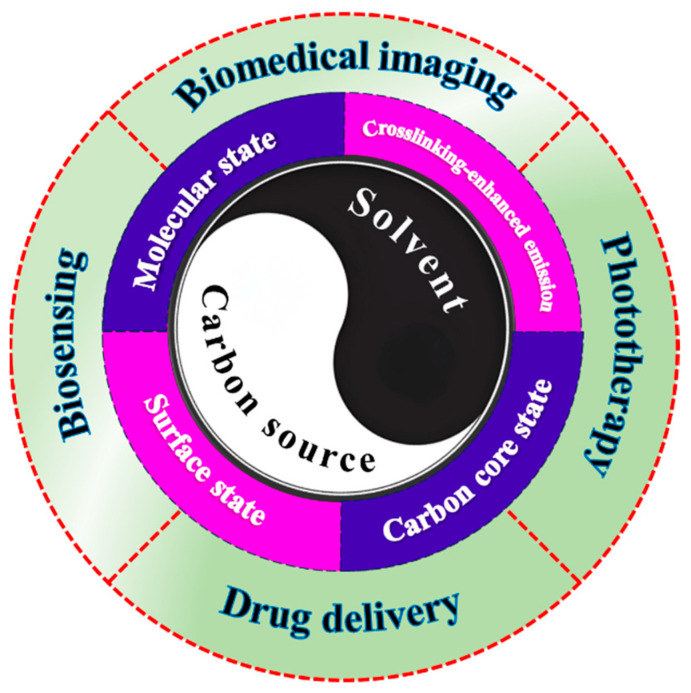
Schematic diagram illustrating the precursor materials, luminescence mechanisms, and biomedical applications of R/NIR-CDs.

Extensive experimental studies have shown that the rational selection of carbon sources and solvents plays a significant role in controlling the long-wavelength fluorescence characteristics of CDs. This review aims to systematically analyze the preparation of R/NIR-CDs from the perspectives of carbon source and solvent selection. Firstly, this paper will review the four main luminescence mechanisms of R/NIR-CDs: including carbon core states [44,45,46], surface states [7,47], molecular states [21,48], and cross-linking enhanced emission effects [49,50], and discuss the specific influence of carbon sources and solvents on these mechanisms. Subsequently, this paper will extensively discuss the applications of R/NIR-CDs in areas such as biological imaging, biosensing, phototherapy, etc., focusing on analyzing the actual application effects of these CDs in various fields and how they effectively address the challenges faced by existing technologies. In addition, this paper will also discuss the issues in advancing the biological applications of R/NIR-CDs and propose possible solutions and future development directions based on current research progress. Through this comprehensive and in-depth analysis, this paper aims to provide a clear perspective on the research and application of R/NIR-CDs, guide future scientific exploration, and promote continuous advancement in this field (Figure 1).

## 2. Luminescence Mechanisms of R/NIR-CDs

Due to the diversity of carbon core states and surface functional groups, the intrinsic structure of CDs is actually quite complex, leading to the uncertainty of the R/NIR luminescence mechanism of CDs. Currently, there is a lack of research specifically correlating the luminescence mechanisms of R/NIR-CDs with the carbon source or solvent used in synthesis. This section will analyze various luminescence mechanisms of R/NIR-CDs in detail and correlate them with the carbon source/solvent.

### 2.1. Carbon Core State

**Figure 2:**
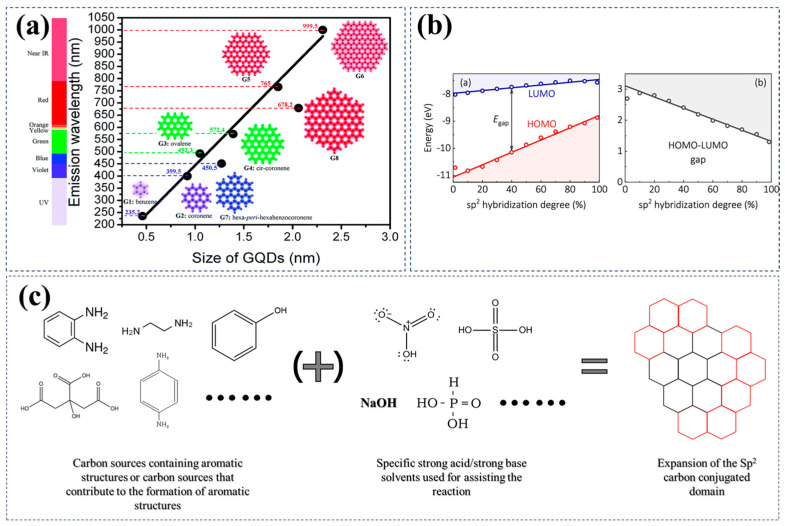
Carbon core state emission mechanism of CDs and its relationship with carbon source/solvent. (**a**) Calculated emission wavelength (nm) using the TDDFT method in vacuum as a function of the diameter of GQDs. The solid line represents the linear fitting of zigzag-edged GQDs. The indicated diameter is the average of the horizontal and vertical dimensions. Reproduced with permission [51]. Copyright 2014, The Royal Society of Chemistry. (**b**) Variation in the size of the LUMO−HOMO gap with different degrees of sp^2^ hybridization. Reproduced with permission [57]. Copyright 2019, American Chemical Society. (**c**) Influence of selected carbon source/solvent on the carbon core state of CDs.

Mechanism Principle: CDs, as a type of quantum dots, are significantly influenced by the quantum size effect in their optical properties (Figure 2a). As the size of CDs decreases, their bandgap widens accordingly, leading to a blueshift in the emission spectrum; conversely, as the size increases, the bandgap narrows, resulting in a redshift [46]. However, an increasing body of research suggests that the term “size” does not solely refer to the actual dimensions of CDs, but rather denotes isolated sp^2^ substructures, namely, effective conjugation lengths. The size of sp^2^ domains in CDs can affect the effective conjugation length, thereby determining the bandgap width of the CDs. R/NIR-CDs can be successfully synthesized by precisely controlling the size of the sp^2^ domains (Figure 2b) [51].

Carbon source/solvent selection: In simple terms, to regulate the carbon core state of CDs, two methods can be selected: indirectly adjusting the size of the sp^2^ domains by controlling the dimensions of CDs, or directly manipulating the size of the sp^2^ domains to control the emission wavelength of the CDs. During the reaction, the carbon source dominates the formation of the carbon core, controlling the carbon core state of the CDs, while the solvent plays an auxiliary role by participating in the reaction process (Figure 2c). In the preparation process of R/NIR-CDs, aromatic compounds such as aromatic amines and phenols are commonly used as carbon sources. These aromatic compounds can form highly π-conjugated systems through self-polymerization during the reaction, which not only enhances the stability of CDs but also effectively extends the absorption wavelength of CDs by decreasing the bandgap. In solvent selection, strong acids and alkalis such as H_2_SO_4_, HNO_3_, and NaOH can promote the dehydration and carbonization processes of carbon sources during the reaction, thereby increasing the carbon core size and further promoting the redshift of emission wavelength.

**Figure 3:**
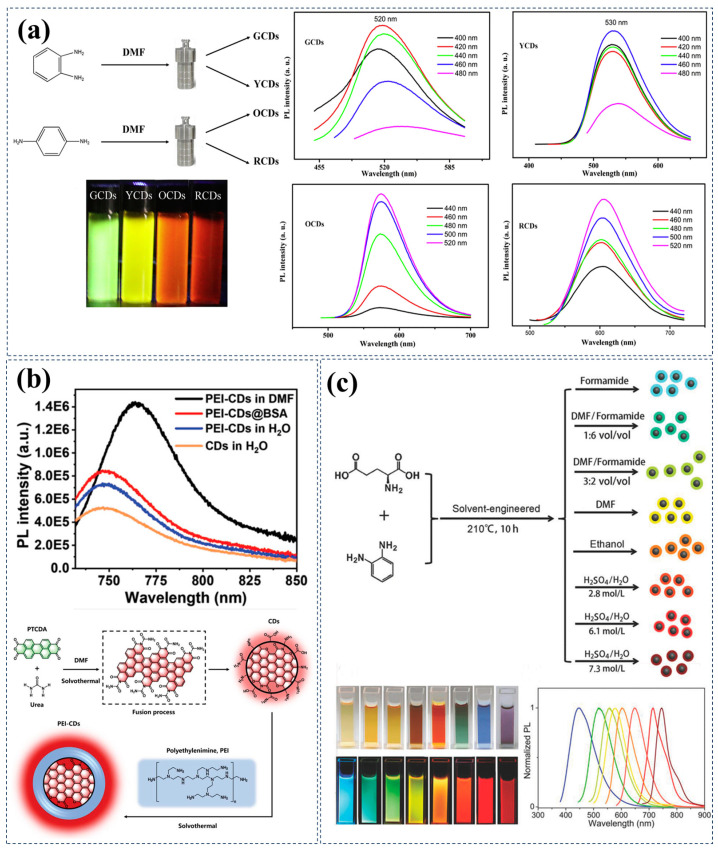
Examples of tuning the carbon core state for red/near-infrared emission by selecting carbon sources or solvents. (**a**) Using o-phenylenediamine or p-phenylenediamine as carbon sources and DMF as a solvent to control the carbon core state through quantum size effects and doping, including a schematic diagram of carbon dot synthesis, photographs of four samples under UV light, and PL spectra of GCDs, YCDs, OCDs, and RCDs under different excitation wavelengths. Reproduced with permission [52]. Copyright 2018, Elsevier. (**b**) Using PTCDA and urea as carbon sources to control the carbon core state by controlling the conjugated system; (**above**) PL spectra of CDs, PEI-CDs, and PEI-CDs@BSA in aqueous solution, as well as the PL spectra of PEI-CDs in DMF solution under 725 nm excitation; (**below**) possible formation processes of CDs and PEI-CDs. Reproduced with permission [55]. Copyright 2022, Wiley-VCH. (**c**) Solvent-engineered synthesis strategy for multicolor fluorescent CDs using l-glutamic acid and p-phenylenediamine as starting materials, controlling the carbon core state through quantum size effects. Photographs of CDs under sunlight and UV light, and normalized PL emission spectra of samples excited at 365 nm. Reproduced with permission [58]. Copyright 2018, Wiley-VCH.

Carbon Source Regulation: In 2018, Sai Lin et al. selected o-phenylenediamine or p-phenylenediamine as carbon sources and used DMF as a solvent to successfully prepare multi-color emitting CDs covering emission wavelengths from green to red using a solvothermal method, with wavelengths ranging from 520 nm to 608 nm [52]. Through an in-depth analysis of TEM images and XPS spectroscopy data, researchers found that the concentration of carbon sources (o-phenylenediamine, p-phenylenediamine) could alter the emission wavelength of CDs, and observed that the size of CDs and N content also varied accordingly (Figure 3a). They speculated that this may be due to the use of aromatic amine carbon sources affecting the quantum size effect and degree of nitrogen doping in CDs, further leading to the red emission characteristics of CDs. Similarly, Liu et al. used o-phenylenediamine (oPD) and aluminum chloride hexahydrate (AlCl_3_·6H_2_O) as precursors to prepare NIR-emitting carbon nanodots (CNDs) with a high photoluminescence quantum yield (PLQY) of up to 57% via a simple in situ solvent-free carbonization method. These CNDs exhibit an emission peak at 700 nm when excited at 532 nm [53]. During the preparation process, with the gradual increase in temperature, aluminum chloride hexahydrate first underwent hydrolysis to generate H_3_O^+^, HCl, and AlO_2_^−^. Subsequently, within oPD, H_3_O^+^ underwent electrophilic reaction with the AlCl_3_ catalyst under high-temperature and -pressure conditions to form polymers. Ultimately, these intermediate state polymers further underwent in situ carbonization accompanied by dehydrogenation reactions, transforming into CNDs with highly efficient aromatic sp^2^ conjugated systems, endowing them with excellent red and near-infrared emission properties.

Structurally similar compounds include phenolic compounds, which typically feature an aromatic ring connected to one or more hydroxyl (-OH) groups. This structure not only imparts inherent aromatic characteristics but also allows phenolic hydroxyl groups to participate in dehydration condensation reactions, thereby enhancing conjugation and expanding the π system. This process can significantly alter the band structure of CDs, rendering them with narrow bandgap characteristics, resulting in fluorescence from red to NIR wavelengths. Zheng et al. used 2,7-dihydroxynaphthalene, citric acid, and lysine as carbon sources to synthesize red fluorescent CDs with solvent-polarity-sensing capabilities via a solvothermal method [52]. Experimental results showed that in the low polarity solvent of anhydrous 1,4-dioxane, the emission wavelength of the CDs was approximately 541 nm. However, with increasing solvent polarity, the emission wavelength of CDs exhibited a significant redshift, especially when water was used as the solvent, reaching approximately 640 nm. After in-depth analysis, researchers attributed the excellent water solubility and unique red fluorescence emission characteristics of CDs mainly to their internal large sp^2^ hybridized conjugated system and abundant surface functional groups. Specifically, the carbon core structure of CDs inherits the conjugated characteristics of naphthol, endowing them with red light emission capability. Meanwhile, the introduction of citric acid and lysine not only helps in forming various surface functional groups (such as amino, hydroxyl, carboxyl, and methylthio groups) but also achieves N and S element doping. These factors collectively enhance the fluorescence stability and QY of CDs and promote their long-wavelength emission characteristics.

In addition to common aromatic compounds, the utilization of polymers as carbon sources, through the regulation of carbon core states, has emerged as an effective approach to achieving red/near-infrared emission. These synthetically engineered polymers, synthesized under carefully designed specific conditions, possess well-defined repetitive structures, thereby imparting specific, meticulously designed characteristics to CDs. According to theoretical calculations, increasing the diameter of the conjugated sp^2^ domain to approximately 2 nm can adjust their bandgap to the near-infrared region [54], yet most commonly used CDs precursors are non-conjugated or possess low conjugation. Consequently, the research group led by Yupeng Liu conceived the idea of using carbon sources with larger conjugated domains to construct CDs with extensive conjugated structures, thereby realizing the preparation of strong NIR-emitting CDs [55]. They ingeniously selected 3,4,9,10-perylene tetracarboxylic dianhydride (PTCDA) with a larger conjugated domain as the carbon source, and successfully synthesized CDs with near-infrared emission characteristics through the solvothermal reaction between PTCDA and urea molecules. It is noteworthy that these CDs exhibit a main absorption peak in water solution at 720 nm, with an emission center at 745 nm, marking the first achievement of CDs simultaneously possessing main absorption and emission peaks in the near-infrared range in aqueous solution. The research group extensively explored the potential of PTCDA as a carbon source. Under solvothermal conditions, PTCDA and urea molecules covalently fused to form rigid graphene-like sheets with extended π-conjugated systems, a structural characteristic that significantly promotes NIR emission of CDs. Subsequently, they further enhanced the NIR emission efficiency in water solution by introducing polyethyleneimine (PEI) through solvothermal treatment. The introduction of PEI molecules not only modified the surface of CDs but also effectively prevented the interaction between water molecules and their conjugated carbon cores, thereby significantly improving the near-infrared emission efficiency in water solution. Furthermore, to further enhance the stability and biocompatibility of CDs, the research group creatively chose the widely used biocompatible protein BSA to combine with PEI-CDs. BSA molecules tightly adsorb onto the surface of PEI-CDs, forming a protective layer that further prevents the quenching effect of water molecules on fluorescence (Figure 3b). The strategy proposed by this research group, utilizing large conjugated polymer carbon sources, holds inspirational significance for the development of NIR-CDs and the promotion of their biomedical imaging applications.

In addition to the artificial carbon sources mentioned earlier, there are many natural carbon sources in nature that can be used to produce R/NIR-CDs, which can also achieve R/NIR emission by regulating the carbon core states. It is worth mentioning that CDs prepared from biomass are not only economically efficient but also environmentally sustainable, possessing excellent water solubility, high biocompatibility, and low cytotoxicity, which provide broad prospects for practical applications [56]. Junjun Liu and his team successfully prepared deep red-emitting carbon dots (CPDs) by using Taxus leaves as the carbon source [4]. These CPDs not only have a narrow full width at half-maximum (FWHM) but also exhibit excellent characteristics of high QY. Through calcination treatment of CPDs and comparative experiments, the research group revealed the mechanism behind these unique properties of CPDs: the π-conjugated system formed by N-heterocycles and aromatic rings effectively controls the single-photoluminescence (PL) centers. In addition to their excellent absorption and emission properties within the deep-red spectral region, CPDs exhibit low cytotoxicity and favorable biocompatibility characteristics. These properties make CPDs ideal probes for both single-photon and two-photon bioimaging. More importantly, CPDs exhibit excellent distribution and excretion in the body, being rapidly cleared through the renal and hepatobiliary systems, ensuring their safety in biological applications. Furthermore, CPDs’ surfaces are rich in functional groups such as amides and hydroxyl groups, allowing them to be easily modified by hydrophilic polymers or small molecules. This modification can improve the water solubility of CPDs and further enhance their biocompatibility, providing more possibilities for their future biomedical applications.

Solvent Regulation: During the preparation of CDs, the use of strong acid or base solvents can effectively modulate the carbon core states, thereby achieving a redshift in emission wavelengths. In a study, Xiong et al. used L-glutamic acid and o-phenylenediamine as carbon sources to successfully prepare NIR-CDs in acidic aqueous solution [58]. As the sulfuric acid concentration increased from 0.06 mol/L to 8.2 mol/L, the dehydration and carbonization reactions of the carbon source were significantly intensified, leading to an increase in carbon dot size, an elevation in graphitic nitrogen content, and a noticeable redshift in fluorescence emission wavelength from 581 nm to 756 nm (Figure 3c). This change not only demonstrated the potential of aromatic amines in the preparation of R/NIR-CDs but also revealed the positive effect of high-concentration acidic solvents in promoting the formation of large-sized carbon cores, thereby achieving emission wavelength redshift. Similarly, using strong alkaline solvents can also achieve similar effects. In the study by Lan et al., by adjusting the concentration of sodium hydroxide (NaOH) aqueous solution and using poly-3-thiopheneacetic acid as the carbon source, carbon nanoparticles (CNPs) with different emission wavelengths were successfully prepared [59]. At higher NaOH concentrations, the C=C component in CNPs decreased, the degree of surface oxidation of CNPs increased, and more surface defects were induced on CNPs, resulting in fluorescence redshift. This study further confirms that under strong acid or base environments, the dehydration and carbonization reactions between carbon sources are more active, thereby promoting an increase in carbon particle size and redshift in fluorescence emission.

In addition to strong acids and bases, the use of other solvents can also promote the formation of carbon cores [60]. In 2022, Xin Yang et al. utilized citric acid as a carbon source and chose DMF as the solvent to successfully prepare two types of CDs with different emission wavelengths via a solvothermal method: CA-BCDs emitting blue light at 423 nm and CA-RCDs emitting red light at 667 nm [61]. It is worth noting that ammonia solution (NH_3_·H_2_O) was specifically added as a solvent during the preparation of CA-RCDs, while it was not added during the preparation of CA-BCDs. The research team conducted an in-depth analysis of this phenomenon and proposed a possible explanation. They believed that the addition of ammonia solution promoted the aromatization process, leading to a wavelength redshift phenomenon in the emission spectrum of CA-RCDs. Specifically, the intermolecular interactions between citric acid and ammonia solution may have facilitated cyclization reactions, thereby constructing larger conjugated domains within the CDs, ultimately resulting in the redshift of the emission wavelength. This discovery not only reveals the significant role of solvents in the preparation of CDs but also provides new insights into regulating the luminescent properties of CDs.

### 2.2. Surface State

**Figure 4:**
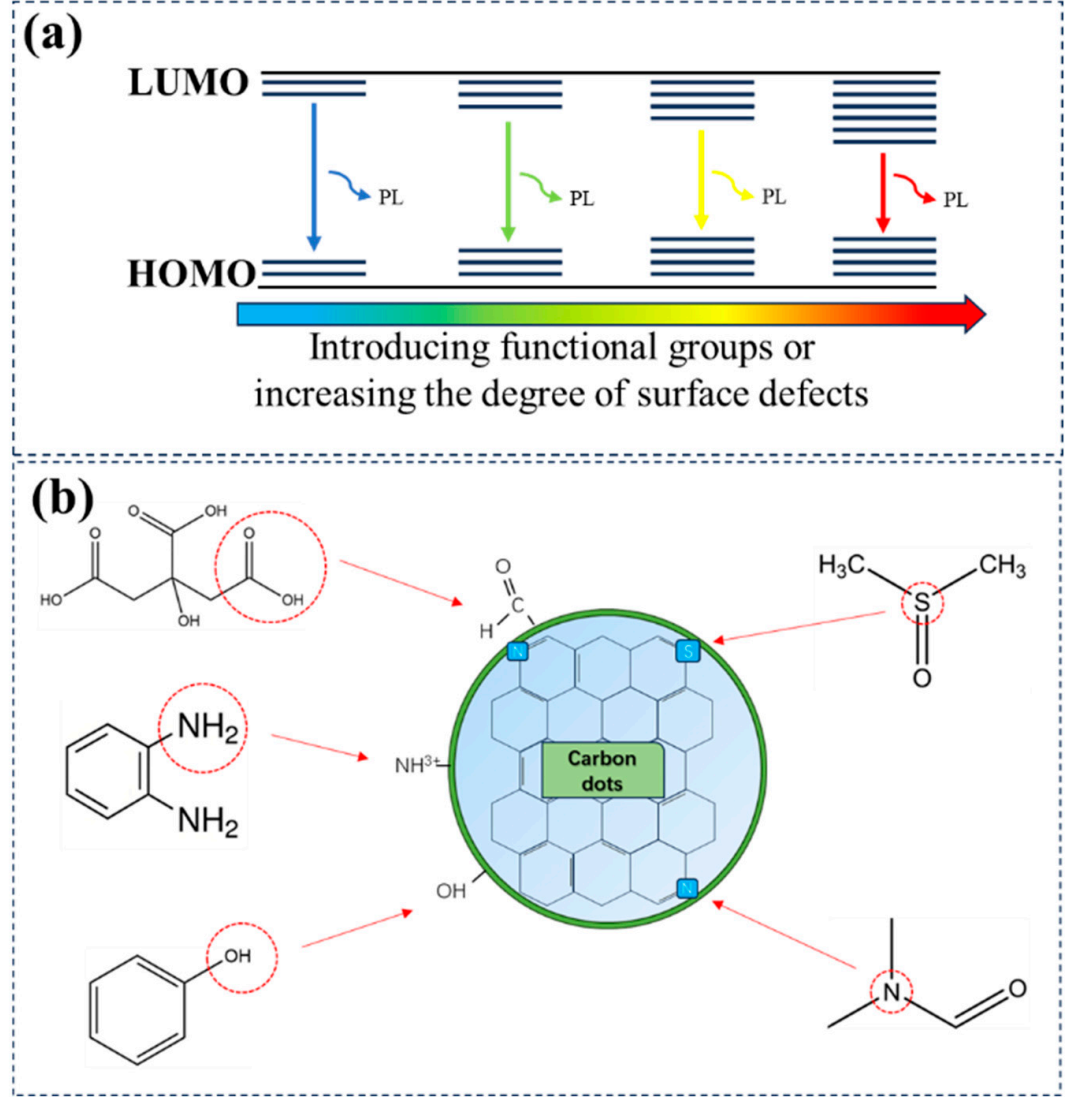
Surface state luminescence mechanism of CDs and its relationship with carbon sources/solvents. (**a**) Schematic diagram illustrating the influence of surface states on the bandgap; (**b**) potential effects of some carbon sources and solvents on the surface states of CDs during the reaction process.

Mechanism principle: CDs have highly complex surface functional groups, and different functional groups can generate multiple unique surface emission sites. With changes in the excitation light, different emission sites on the surface of CDs dominate the emission, causing the emitted light to vary accordingly. The regulation of surface states involves introducing specific functional groups (or heteroatoms) to increase the surface defects or degree of surface oxidation of CDs (Figure 4a). These defects can function as traps for excitons and introduce new energy levels, consequently narrowing the energy gap between the Highest Occupied Molecular Orbital (HOMO) and the Lowest Unoccupied Molecular Orbital (LUMO), leading to a redshift in the emission wavelength [5].

Carbon source/solvent selection: The choice of carbon source and solvent significantly influences the functional groups and doping elements on the surface of CDs, which, in turn, affect the electronic structure and energy levels of CDs, thereby controlling the emission wavelength. To achieve a redshift in emission wavelength, it is advisable to select carbon sources and solvents containing specific elements or functional groups (Figure 4b). When selecting carbon sources, compounds that can provide doping with elements such as N, S, O or possess functional groups such as amino and carboxyl groups should be given priority. Similarly, solvents such as DMF, DMSO, PVP, and NMP are all excellent choices for preparing R/NIR-CDs. They can introduce doping during the reaction process, alter the surface states of CDs, and insert new energy levels into excited and ground-state orbitals, resulting in emission wavelengths of the resulting CDs closer to the NIR region.

Carbon source regulation: Xiang Miao et al. utilized citric acid and urea as precursors and successfully synthesized CDs with multicolor luminescent properties via a solvothermal method [6]. They observed that when the molar ratio of citric acid to urea ranged from 0.3 to 0.4, a significant redshift in the emission wavelength of CDs from the blue to red region occurred. Simultaneously, X-ray photoelectron spectroscopy (XPS) analysis revealed a synchronous increase in the carboxyl group (-COOH) content with the redshift of the emission wavelength. This finding suggests that the presence of citric acid at specific ratios effectively introduces carboxyl groups on the surface of CDs, thereby increasing the interlayer distance and altering the band structure, consequently achieving a remarkable redshift in the emission wavelength. Additionally, the analysis indicates that the polymerization reaction of citric acid and urea is essentially an acid-catalyzed process [62]. Therefore, a precise analysis of the amount of citric acid used may provide an effective approach to modulating surface states, potentially offering insights into the influence on optical properties.

**Figure 5:**
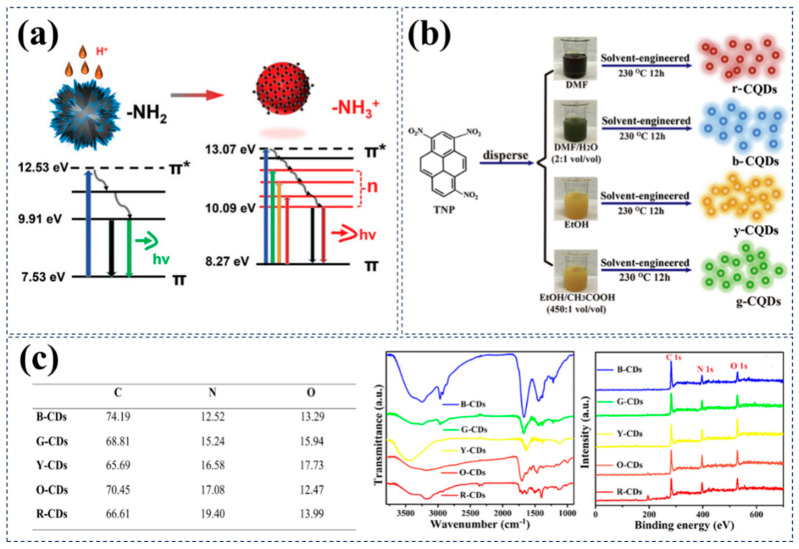
Examples of adjusting surface state red/near-infrared luminescence through the selection of carbon sources or solvents. (**a**) Control of surface states by acidifying o-phenylenediamine to change functional groups. The introduction of activated aromatic amino (-NH_2_) groups on Red-CDs introduces numerous n energy orbital levels, narrowing the bandgap of excited state energy transitions, thereby triggering red fluorescence emission. Reproduced with permission [63]. Copyright 2023, Wiley-VCH. (**b**) Surface state modulation through doping using TNP as the carbon source, with DMF or EtOH as the sole or major solvent, and H_2_O or CH_3_COOH as auxiliary solvents. Reproduced with permission [68]. Copyright 2018, Elsevier. (**c**) Surface state modulation by doping using 1,2,4-triaminobenzene hydrochloride as a precursor, with water, ethanol, tetrahydrofuran, ethyl acetate, and acetone as solvents. (**Left**) C, N, O contents of the five samples; (**Right**) FTIR and XPS spectra of B-CDs, G-CDs, Y-CDs, O-CDs, and R-CDs. Reproduced with permission [69]. Copyright 2020, American Chemical Society.

On the other hand, aromatic amines are widely employed in the preparation of CDs with tunable luminescence through surface state regulation. For instance, in 2022, Qing Zhang et al. successfully synthesized red-emitting CDs capable of both single- and two-photon excitation by acidifying o-phenylenediamine [63]. Combining analysis from UV–vis absorption, fluorescence (FL) emission, FTIR, NMR, Zeta potential, and UPS, researchers revealed that protonation of the pyridinic nitrogen (-N=) structure of conjugated amino groups narrowed the bandgap of photon transitions, ultimately resulting in near-infrared fluorescence emission of the CDs (Figure 5a). Furthermore, deprotonation of the red-emitting CDs led to the disappearance of the long-wavelength absorption peak, while switching to graphite or pyrrole nitrogen-doped CDs maintained the fluorescence emission wavelength or induced a blue shift, further validating the researchers’ conclusions. In addition to functional groups, doping-induced alterations in surface-state luminescence of CDs are also a common method for preparing R/NIR-CDs. In another study, Qiaoqiao Ci et al. developed a novel type of near-infrared second window (NIR-II) fluorescent iron-doped CDs (Fe-CDs), which exhibited high QY [64]. The research team employed a simple one-pot hydrothermal synthesis strategy using hydrochloric acid dopamine (DA) and oPD as carbon sources, simultaneously utilizing FeCl_3_·6H_2_O as a dopant. These Fe-CDs demonstrated excellent stability in acidic solutions, with peak absorption and emission wavelengths reaching up to 830 nm and 1000 nm, respectively. Moreover, these CDs have been successfully applied in the non-invasive real-time monitoring of gastric pH during mouse food digestion, showcasing their immense potential in biological imaging. XPS analysis further confirmed previous findings, indicating that a higher proportion of C=O and graphitic N structures in CDs could lead to a redshift in fluorescence emission [65]. More importantly, this study revealed a new significant finding through comparative experiments: the introduction of Fe^3+^ could promote the formation of C=O and graphitic-N structures from carbon sources like oPD, significantly enhancing the fluorescence emission of Fe-CDs in the NIR-II region. This discovery provides new insights and strategies for designing and preparing CDs with near-infrared fluorescence properties.

Solvent regulation: Solvents also play an irreplaceable role in regulating the surface state luminescence of CDs. In 2018, Qu et al. utilized citric acid and urea as carbon sources, with dimethyl sulfoxide (DMSO) as the solvent, and processed them at 160 °C for 4 h using a solvothermal method, resulting in sulfur and nitrogen co-doped CDs [66]. These CDs, when excited by a 655 nm laser, emit light with a central wavelength at 720nm, with a high photothermal conversion efficiency of up to 59%. DMSO provides doping of sulfur and nitrogen elements during the formation of the carbon core structure, which affects the electronic and band structures of the CDs. Studies have shown that sulfur doping decreases the optical bandgap of CDs, leading to a redshift in the emission wavelength, consistent with previous reports [9].

Although the pH of the solvent also has a significant influence on the optical properties of CDs, it is not just the pH value of the solvent that needs to be considered when selecting a solvent. The research group led by Bai Yang used oPD as the carbon source, with HNO_3_ as the solvent, to prepare strong red fluorescent CDs (630 nm) via a one-step hydrothermal method [67]. The researchers found that substituting the acid in the solvent with other acids such as H_2_SO_4_ and H_3_PO_4_ could not achieve the preparation of red fluorescent CDs. This indicates that the joint action of H^+^ ions and NO^3−^ ions is a key factor in achieving efficient red-light emission.

To specifically analyze how solvent selection influences the emission characteristics of CDs, researchers conducted a series of studies while keeping the carbon source constant and adjusting other variables. For instance, Jing Zhan et al. employed TNP molecules as the carbon source and utilized four different solvent schemes: pure DMF solvent, pure EtOH aqueous solvent, DMF–water mixture, and EtOH–acetic acid mixture [68]. By changing only the solvent, they successfully prepared CQDs with different emission wavelengths (Figure 5b). In these experiments, CDs prepared using pure DMF solvent exhibited the longest emission wavelength, with the emission peak at 620 nm, while the CDs from the DMF–water mixture group had the shortest emission wavelength, with the emission peak at 460 nm. The researchers believe that the variation in emission wavelength of these CDs may stem from both changes in the bandgap of the CDs and the luminescence of surface states. Specifically, when DMF serves as the solvent, its strong solvation ability promotes the extensive fusion of TNP molecules, leading to larger CQDs (redshift in emission wavelength). Additionally, DMF may further alter the surface states of CDs through nitrogen doping, affecting their optical properties. On the other hand, the blueshift in emission wavelength of CDs when DMF is mixed with water as the solvent could be attributed to the weaker ability of protonated solvents compared to non-protonated solvents in promoting carbonization and dehydration reactions, thereby resulting in a blueshift in emission wavelength. Kumari et al. conducted similar studies, using 1,2,4-triaminobenzene dihydrochloride as a precursor and selecting water, ethanol, tetrahydrofuran, ethyl acetate, and acetone as solvents, to prepare a series of CDs with different emission wavelengths [69]. During the spectral characterization of the synthesized CDs, the research team observed a redshift phenomenon in the emission spectra of CDs with an increase in the nitrogen content in the solvent (Figure 5c). This phenomenon can be attributed to the introduction of new energy levels by nitrogen doping, leading to a reduction in the bandgap and thus a redshift in emission wavelength. Further elemental analysis results showed an increasing trend in the content of C-O bonds from blue CDs to orange CDs, further confirming that using different solvents can induce a redshift in emission wavelength by affecting surface states.

### 2.3. Molecular State

**Figure 6:**
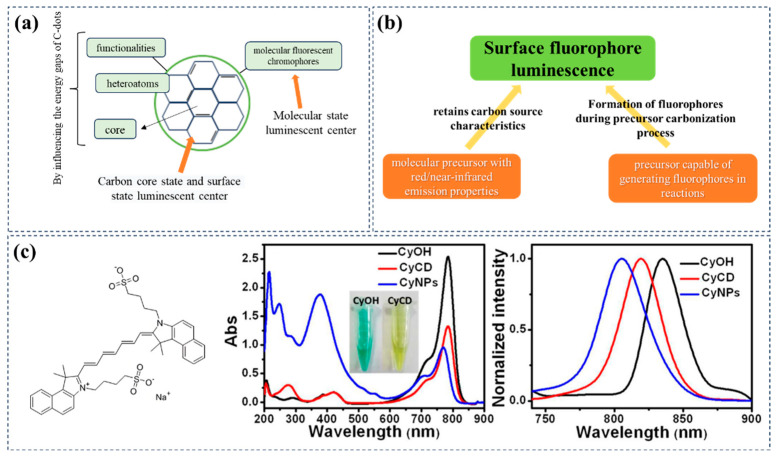
The molecular emission mechanism of carbon dots and its relationship with carbon sources/solvents. (**a**) Schematic diagram of molecular state emission; (**b**) common methods for preparing CDs with molecular state emission; (**c**) taking indocyanine green as an example, Zheng et al. used indocyanine green as the carbon source and PEG800 as the solvent to prepare molecular state emitting CDs CyCD; the figure shows the UV–visible absorption spectra and photoluminescence spectra of the precursor CyOH, CDs, CyCD and CyNPs synthesized without adding PEG800 in ethanol. Reproduced with permission [75]. Copyright 2016, American Chemical Society.

Mechanism principle: During the carbonization of carbon dot precursors, molecular fluorophores form and attach to the surface of CDs, becoming their main fluorescence emitters. These fluorophores are capable of inheriting and retaining the characteristics of the carbon precursor, thereby imparting similar optical properties to the CDs (Figure 6a). Existing research has confirmed that the molecular fluorophores on the surface of CDs play a crucial role in the emission process, and the excitation-dependent emission characteristics provide reliable indicators for revealing the contributions of different molecular fluorophores [70].

Carbon source/solvent selection: Generally, to controllably prepare molecular-state luminescent R/NIR-CDs, it is necessary to design the required surface fluorophores from the precursor stage. This can be achieved in two ways: by designing the carbon source to naturally generate red/near-infrared molecular fluorophores during the reaction process or by directly selecting dyes with the desired properties as carbon sources and partially controlling the reaction conditions to prepare R/NIR-CDs with similar optical properties (Figure 6b). The main advantage of this method is that it allows for the attainment of the advantages of CDs while retaining the properties of the dye (or other fluorophores) itself.

**Figure 7:**
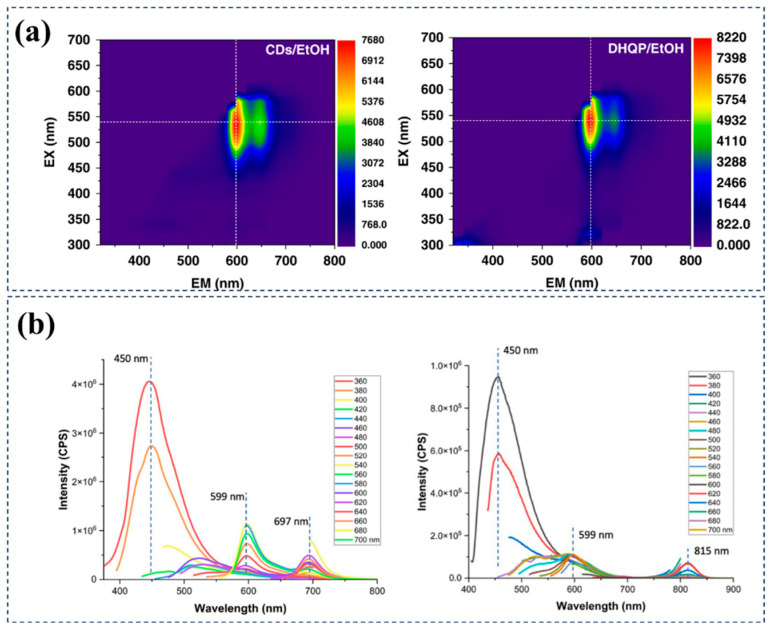
Examples of molecular-state red/near-infrared emission regulation achieved by carbon source or solvent selection. (**a**) Molecular-state-dominant red-emitting CDs prepared using o-phenylenediamine (oPD) and catechol (CAT) as carbon sources; CDs (**left**) and DHQP (**right**) in ethanol solution, excitation emission spectra. Reproduced with permission [72]. Copyright 2022, Springer Nature. (**b**) R-CDs prepared using citric acid, ICG, and PEG1000 as precursors; (**left**) R-CD PL spectrum, (**right**) ICG PL spectrum. Reproduced with permission [74]. Copyright 2023, Elsevier.

Carbon source/solvent regulation: Xin Geng et al. utilized microwave-assisted synthesis, employing variously substituted meta-aminophenols and citric acid as precursors. By solely adjusting the substitution positions of meta-aminophenols, they successfully synthesized a series of multicolor MitoTCDs [71]. These MitoTCDs exhibit tunable fluorescence ranging from green to red with high QYs, demonstrating excellent performance in HeLa mitochondria-targeting imaging and long-term stable tracking of cell passages, making them suitable for prolonged cellular imaging studies. During the synthesis, the researchers prepared diphenyl ether derivative molecules through in situ condensation of meta-aminophenols and their derivatives. Subsequently, these molecules underwent Friedel–Crafts reactions with carboxylic acid derivatives to generate rhodamine fluorophores, imparting unique fluorescence properties to MitoTCDs. In 2022, the Sun group efficiently synthesized red-emitting CDs using o-phenylenediamine and catechol (CAT) as raw materials [72]. To further investigate the optical properties of CDs, the researchers conducted comparative studies with commercial dyes, exploring the effects of solvents, pH, and time-resolved PL on the optical properties of CDs and DHQP. Through comparison, they found that the properties of CDs were extremely similar to DHQP, confirming CDs as molecular-state-driven emitters with their molecular fluorophores being DHQP. (Figure 7a). Furthermore, the Sun group conducted in-depth studies on the mechanism of red-emitting CDs production. They found that oPD and CAT initially reacted to form intermediate products (2,3-DAPN and quinone). Subsequently, these intermediates further reacted with oPD to generate DHQP molecules. As the reaction temperature increased and the time prolonged, the molecules gradually grew and carbonized, forming DHQP molecules embedded or linked with single-layer graphene. These DHQP molecules were connected to CDs through sp^3^ bonds, while the benzene rings at the edges of DHQP molecules were merged into the structure of conjugated CDs nuclei. Eventually, these single-layer graphene stacked into zero-dimensional CDs, thereby achieving the preparation of red-emitting CDs.

Similarly, Pang et al. directly utilized long-wavelength-emitting organic fluorophores as carbon sources to prepare R/NIR-CDs via a hydrothermal method [73]. They chose the biological dye azure A chloride and copper gluconate rich in hydroxyl functional groups as carbon sources, successfully synthesizing NIR-CDs (λ_ex_ = 658 nm; λ_em_ = 683 nm) with near-infrared emission characteristics. The optical properties of the dye were largely preserved. In 2023, Ferreira et al. obtained R-CDs with red light emission by treating citric acid, ICG, and PEG1000 using ultrasound and microwave methods [74]. By comparing the emission spectra of R-CDs and ICG, the researchers found that besides exhibiting red emission at 697 nm, R-CDs also had an emission peak at 599 nm highly similar to ICG (Figure 7b), showing high consistency in their visible light spectra. Therefore, they speculated that the electronic structure of ICG molecules was partially retained in R-CDs, forming an effective linkage with CDs through PEG chains. Further FTIR characterization studies confirmed that R-CDs retained most of the functions of ICG, and the molecular fluorescence group formed by ICG played a dominant role in the red-light emission of R-CDs.

### 2.4. Cross-Linking Enhanced Emission

Mechanism principle: The cross-linking enhanced emission (CEE) effect was first proposed by Yang et al. in 2014 [76]. In simple terms, the CEE effect refers to the effective restriction of fluorophore vibration and rotation through cross-linking within the polymer framework of CDs, thereby inhibiting non-radiative transition processes. This mechanism not only enhances the luminescence intensity of existing fluorophores but also enables the re-emission of potential fluorophores that were originally unable to emit light due to non-radiative transitions. In carbonized polymer dots, the reduction in fluorophore distance caused by the CEE effect also leads to electron cloud overlap and level coupling splitting, further reducing the bandgap, resulting in redshifted emission wavelengths [77]. In summary, the main effects of the CEE effect are manifested in three aspects: firstly, it activates potential molecular fluorophores, enabling them to emit light; secondly, by adjusting the emission wavelength of CDs, it alters their optical properties; finally, it significantly enhances the PLQY of CDs. Essentially, the CEE effect, through cross-linking, exerts a fixed effect, profoundly impacting the optical properties of CDs through bonding mechanisms.

**Figure 8:**
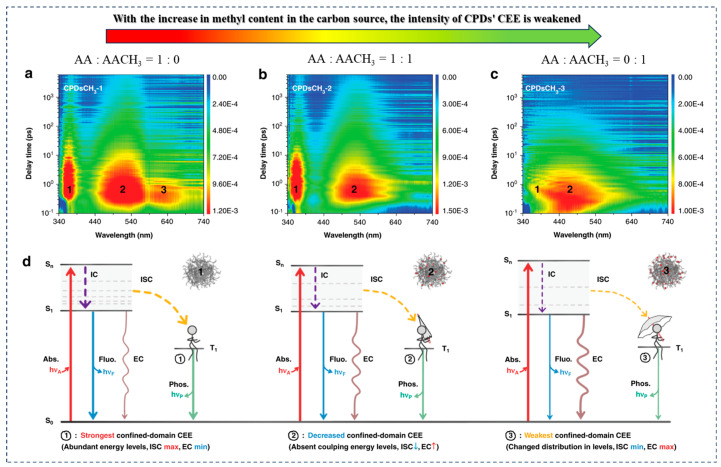
Spatial interactions observed within CPDs (Confinement-Enhanced Emission, CEE). Two-dimensional pseudocolor TA maps of CPDs with increasing methyl content ((**a**): CPDsCH3-1, (**b**): CPDsCH3-2, (**c**): CPDsCH3-3) (excitation wavelength 320 nm, detection wavelength 340–740 nm, scan delay 0.1 ps–6 ns). (**d**): The impact of three-dimensional confined CEE on the energy levels of CPDs). Reproduced with permission [77]. Copyright 2022, Springer Nature.

**Figure 9:**
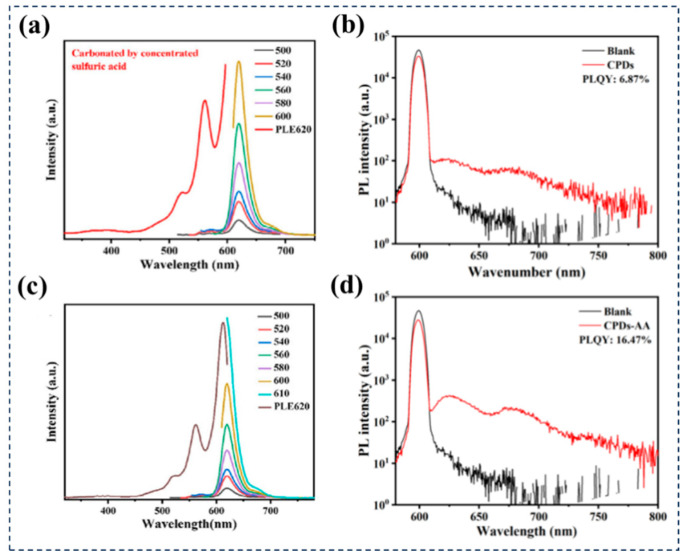
CPDs with red emission and CPDs-AA were successfully prepared using a room-temperature (RT) synthesis method with ortho-phenylenediamine as the carbon source. (**a**) PL and PLE spectra of CPDs. (**c**) PL and PLE spectra of CPDs-AA. (**b**) Photoluminescence quantum yield of CPDs. (**d**) PLQY of CPDs-AA. The black lines represent spectra without samples in the integrating sphere. Reproduced with permission [78]. Copyright 2023, Elsevier.

Carbon source/solvent regulation: In order to clearly define the concept of confined environment effect in carbonized polymer dots, the research group led by Yang conducted a series of experiments [77]. They used acrylic acid and methyl acrylic acid in different ratios as carbon sources to control the polymerization degree of CPDs and systematically studied the influence of CEE on the optical properties of CPDs. By employing advanced femtosecond transient absorption (TA) spectroscopy, the researchers obtained information on the excited state structure of CPDs. They found that increasing the methyl content in the carbon source weakened the intensity of CEE within the CDs, manifested by the disappearance and shift of partial emission peaks, as well as a decrease in luminescence intensity. These experimental results not only confirmed the critical role of CEE in the luminescence process of CPDs but also provided important evidence for utilizing CEE to regulate and optimize the optical performance of CPDs (Figure 8). Sun et al. utilized a room temperature (RT) synthesis method with ortho-phenylenediamine as the carbon source to prepare precursors through a polymerization process, followed by manual carbonization with concentrated sulfuric acid, successfully obtaining CPDs with red emission [78]. The researchers believed that these CPDs shared a common molecular fluorophore with the intermediate product PDs, namely 2,3-DAPN. Considering the separateness of the polymerization and carbonization steps, Sun et al. attempted to introduce acetic acid (AA) during the polymerization stage to optimize the fluorescence properties of CPDs, resulting in the synthesis of a new type of CPDs-AA. Although CPDs-AA exhibited consistency with the original CPDs in absorption and emission curves, the PLQY of CPDs-AA reached 16.47%, significantly higher than the 6.87% of CPDs, attributed to the cross-linking enhanced emission effect (Figure 9). TGA and DTG curve analysis revealed a large amount of polymer chains within CPDs-AA, which can provide numerous donor and acceptor sites for hydrogen bonding, thereby enhancing PLQY through the CEE effect. This discovery not only elucidates the optimization mechanism of CPDs’ fluorescence performance but also provides a new strategy for improving the optical properties of CPDs by regulating carbon sources and solvents. Similarly, Tao et al. synthesized novel polymer CDs (PCDs) with a QY as high as 44.18% using polyacrylic acid (PAA) and ethylenediamine as carbon sources through a one-step hydrothermal method [79]. The researchers found that PCDs and the hydrothermal product of EDA (EDA-H) exhibited very similar optical properties, indicating that the fluorophores of EDA-H-like compounds may be the main emission source of PCDs, i.e., the emission of PCDs originates from the molecular state. It is noteworthy that although PCDs and EDA-H have similar fluorophores, the QY of PCDs is significantly higher than that of EDA-H, and their emission wavelengths also undergo a redshift. This phenomenon is likely due to the formation of cross-linking structures by PAA during the hydrothermal reaction process, which effectively immobilizes the fluorophores, enhances their emission intensity, and shifts the emission wavelength by altering the chemical environment of the fluorophores.

### 2.5. Summary

The luminescent properties of CDs can be tuned to achieve red/near-infrared emission by regulating their carbon core, surface, and molecular states. Effective strategies include carefully selecting carbon sources with large conjugated domains and solvents with specific structures, utilizing doping with heteroatoms and functional groups, and designing carbon sources with specific optical properties. Additionally, CEE optimizes the luminescent performance of CDs by immobilizing fluorophores and optimizing precursor compositions. In practical applications, a single mechanism often fails to adequately explain the optical properties of CDs, as they are typically governed by a combination of these mechanisms. However, it is still possible to identify the dominant luminescent mechanism through an analysis of the data. This paper attempts to analyze the primary mechanisms responsible for the R/NIR luminescence of CDs based on the referenced literature and summarizes them in Table 1.

As can be seen from the data in Table 1, CDs with surface states as the main mechanism have more potential in achieving near-infrared region I luminescence, while CDs mainly relying on carbon core states tend to emit red light. This indicates that when researchers prepare NIR-CDs, they should not only utilize the carbon core state to adjust the emission band to the red region, but also give more consideration to adding specific surface functional groups or specific dopants to the CDs to utilize the surface state and enable the CDs to obtain longer emission wavelengths. On the other hand, the emission wavelength of CDs with molecular states as the main mechanism depends on the specific molecular fluorophores on the surface of the CDs, which is also an effective preparation method for obtaining CDs with ideal properties.

## 3. Bioapplications of Red/NIR C-Dots

R/NIR-CDs, with their excellent tissue penetration, remarkable water solubility, superior biocompatibility, low toxicity, and outstanding optical properties, have shown tremendous application prospects in the diagnosis and treatment of cancer. Especially in the fields of biomedical imaging and sensing, fluorescence probes based on R/NIR-CDs are gradually becoming indispensable due to their high signal-to-noise ratio and excellent tissue penetration capabilities [102]. Additionally, the unique structural characteristics of CDs enable them to carry drugs in various ways and precisely target specific cells [31]. Careful selection of carbon sources and solvents during the preparation process is crucial for achieving desirable application effects. By optimizing preparation conditions, R/NIR-CDs with specific functionalities and performance can be obtained. This section provides a detailed analysis of the applications of R/NIR-CDs in biomedical imaging, biosensing, phototherapy, targeted drug delivery.

### 3.1. Bioimaging

Biological imaging technology, as a key method for revealing the structure and physiological status of biological tissues, enables real-time monitoring of the internal environment of organisms through non-invasive detectors and probes. The application of this technology in the field of clinical medical diagnosis is gradually becoming a research hotspot, especially in the development of next-generation fluorescent imaging probes, where R/NIR-emitting CDs are highly regarded for their unique properties [102]. There are higher demands on the QY, excitation and emission spectral characteristics, biotoxicity, and photodamage of R/NIR-CDs, aiming for more precise and safer applications in the field of biological imaging. Recently, CDs have shown extensive potential applications in plant tissue imaging, microbial detection, and cellular-level biological imaging. They can not only specifically target organelles but also enable researchers to track and monitor the dynamic changes in biological tissues in real-time, providing powerful tools for life science research and medical diagnosis.

**Figure 10:**
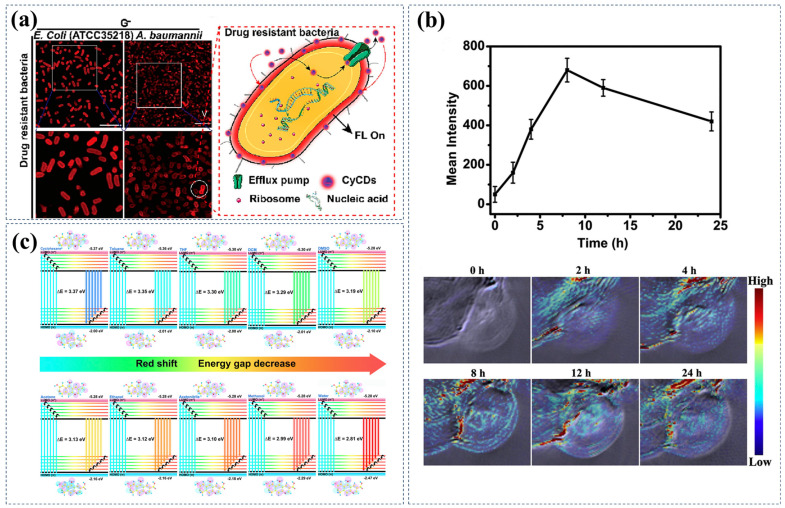
(**a**) Fluorescence imaging of various drug-resistant bacteria (*E. coli* (ATCC35218, G−), *A. baumannii*, and MR *S. aureus*) mixed with CyCDs, as well as the imaging mechanism of CyCDs in drug-resistant bacteria. Reproduced with permission [103]. Copyright 2023, Elsevier. (**b**) In vivo photoacoustic imaging of mice after intravenous injection of HBCDs in PBS and photoacoustic intensities of tumors in the imaging. Reproduced with permission [105]. Copyright 2018, Elsevier. (**c**) HOMO and LUMO energies and orbitals of the optimized structure of the CDs in solvents of different polarities. Reproduced with permission [25]. Copyright 2023, The Royal Society of Chemistry.

Fluorescent imaging: Fluorescence imaging is a real-time imaging technique that provides high resolution and contrast using fluorescent probes. Compared to traditional imaging methods, fluorescence imaging offers advantages such as low cost, simplicity of operation, and lack of radiation. R/NIR-CDs, as a highly promising fluorescent probe, possess a series of advantages. In terms of imaging, their long-wavelength emission enables deeper penetration and imaging unaffected by the spontaneous emission of biological organisms. In terms of safety, the unique graphite core and surface functional groups of CDs result in good biocompatibility and low toxicity, minimizing their impact on biological entities. In terms of cost, the simple preparation method and inexpensive precursor materials make R/NIR-CDs highly suitable for large-scale production. In experiments conducted by Pengli Gao and colleagues, they successfully prepared advanced near-infrared carbon dot fluorescent nanoprobes (NIR-CDs) specifically designed for kidney imaging [81]. These NIR-CDs not only exhibit excellent near-infrared fluorescence characteristics but also demonstrate an extremely small size and excellent biocompatibility, almost without renal toxicity. Owing to their size and biocompatibility, these nanoprobes can efficiently clear from the body by glomerular filtration. On the other hand, WeiJian Liu and colleagues synthesized CyCDs with significant near-infrared imaging capabilities using a solvothermal method, with Cy7-CH_3_ as the carbon source and 3-hydroxytyramine hydrochloride as the surface passivator [103]. The amino groups on the surface of CyCDs give them a positive zeta potential (+0.32 mV), originating from the 3-hydroxytyramine hydrochloride used in the synthesis process. These positively charged amino groups enable CyCDs to rapidly bind to the negative charges on the surface of bacteria, achieving specific recognition of drug-resistant bacteria and effective labeling and imaging of bacteria (Figure 10a). Additionally, the same research group has used Cy-COOH and polyethylene glycol (PEG600) as carbon sources to prepare near-infrared CDs with a maximum emission wavelength of up to 710 nm using a solvothermal method [98]. These CDs are not only suitable for deep tissue imaging but also capable of producing singlet oxygen under light exposure, thus acting as photosensitizers in PDT therapy. From the precursor perspective, the presence of Cy-COOH ensures the near-infrared luminescent properties of the CDs, while the addition of PEG600 enhances their water solubility, making them more suitable for biological applications.

Photoacoustic imaging: Photoacoustic imaging (PA) is an imaging technique that combines optical and ultrasound technologies. It involves the generation of heat in tissues through laser excitation, causing local tissue expansion and contraction, resulting in the generation of sound waves that are detected and converted into images by detectors. The advantage of photoacoustic imaging lies in its combination of the high resolution of optical imaging and the deep penetration capability of ultrasound imaging, allowing for clear imaging of deep tissue. Additionally, due to the low scattering of tissues to ultrasound waves, photoacoustic imaging has greater penetration power than traditional optical imaging, aiding in the early diagnosis of diseases. CDs with strong absorption coefficients in the R/NIR spectrum are particularly suitable as contrast agents in PA imaging, as they efficiently convert light energy into heat [104]. Ji et al. prepared a type of HBCDs using Ganoderma lucidum as a precursor, which exhibited excellent fluorescent properties and photoacoustic imaging capabilities [105]. By injecting HBCDs into live mice and obtaining photoacoustic images of tumors and major organs 24 h post injection and at specific time points, the study demonstrated that HBCDs could effectively accumulate at tumor sites and exhibit higher signal intensity in photoacoustic imaging, indicating their potential in tumor imaging (Figure 10b). In 2022, Han et al. prepared PCDs by mixing urea with citric acid in a DMSO solution using a one-step solvothermal method [106]. PCDs displayed significant photoacoustic signals under 808 nm laser excitation, enabling their use in photoacoustic imaging. By conducting imaging tests on PCDs in vitro and in vivo models, the researchers were able to observe the distribution and penetration of PCDs in different tissues. Particularly in in vivo experiments, photoacoustic imaging of PCDs in tumor tissues was performed using a mouse fluorescence imaging system, revealing gradually enhanced fluorescence from the injection site to the tumor center and distant tumor tissues. Similarly, Xu et al. prepared NIR CDs (supra-CNDs) using citric acid and urea as carbon sources and DMSO as a solvent [107]. Besides possessing absorption and emission peaks at long wavelengths, supra-CNDs could also be used for photoacoustic imaging. Through experiments conducted in mice, the researchers found that supra-CNDs could accumulate in tumor tissues via blood circulation, with the strongest photoacoustic signal in the tumor area observed 5 h after intravenous injection, providing strong contrast signals in photoacoustic imaging.

### 3.2. Biosensing

As a type of fluorescent nanomaterial with broad application prospects, CDs in the near-infrared range, owing to their small size, characteristics allowing for flexible surface modification, and outstanding chemical stability, among other significant advantages, have been widely employed in various in vitro and in vivo sensor devices. The emission characteristics in the R/NIR spectrum, coupled with excellent biocompatibility, make them particularly suitable for applications in biological sensing within organisms.

Zheng Yang et al. synthesized red fluorescent CDs using a solvothermal method, which not only enables the detection of intracellular polarity (Figure 10c) but also exhibits dual targeting capabilities towards mitochondria and lysosomes [25]. The introduction of naphthol ensures the red emission of the CDs, while the presence of cysteine achieves N, S co-doping inside the CDs, enhancing their biocompatibility and functionality. Hanyue Cui et al. synthesized NIR-CDs using a one-step hydrothermal method; these CDs serve as fluorescent probes, displaying high sensitivity and selectivity in detecting ciprofloxacin (CIP), providing a novel approach for drug residue detection [90]. Zhang et al. prepared CDs using sodium sulfate and o-phenylenediamine as precursors through a hydrothermal method, exhibiting high color purity and long-wavelength single-photon fluorescence emission properties, as well as near-infrared-induced two-photon fluorescence emission capabilities [101]. These CDs, when co-cultured with Escherichia coli, can attach to bacteria and emit bright red signals under a fluorescence microscope. Additionally, Xu et al. prepared RCDs using sulfur and citric acid as carbon sources and methanol as a solvent, where the fluorescence intensity increases with temperature and shows a good linear relationship with temperature [108]. This characteristic allows RCDs to infer changes in intracellular temperature by measuring changes in fluorescence intensity, providing a reliable method for studying cellular physiology and disease states. In addition to the sensing applications mentioned above, R/NIR-CDs have also achieved precise measurements of metal ions [109,110,111,112], small biological molecules [113,114], pH values [64], and biomarkers [115].

### 3.3. Phototherapy

Compared to traditional cancer treatment methods, phototherapy has many advantages such as low trauma, high selectivity, no drug resistance, and minimal side effects, making it highly promising in the field of clinical medicine [116]. NIR light, due to its unique characteristics such as deep tissue penetration and low autofluorescence interference, has distinct advantages in cancer diagnosis and treatment. CDs, on the other hand, are well suited as nanotherapeutics due to their excellent biocompatibility, low cytotoxicity, and outstanding photostability. Applying CDs that emit R/NIR light in cancer therapy can fully leverage the advantages of both. Common phototherapy methods include photothermal therapy (PTT) and photodynamic therapy (PDT). In PTT, R/NIR-emitting CDs efficiently convert light energy into heat, which is then used to locally destroy tumor cells. In PDT, these CDs can act as photosensitizers, generating reactive oxygen species under light irradiation, thereby inducing apoptosis or necrosis of tumor cells.

Photothermal Therapy: PTT is a non-invasive method that utilizes light energy converted into heat to treat cancer. By injecting photothermal conversion materials into the tumor site and exposing them to an external light source, heat is generated to kill cancer cells. This therapy is characterized by high selectivity, low toxicity, and short treatment duration [36,117]. The significant advantage of PTT over PDT lies in its independence from the presence of oxygen, making it effective in generating heat in the hypoxic tumor microenvironment, thus showing great potential for treating challenging hypoxic tumors. R/NIR-CDs are used as PTT agents due to their ability to generate reactive oxygen species (ROS), low phototoxicity, high photothermal conversion efficiency (PCE), and deep tissue penetration [118].

**Figure 11:**
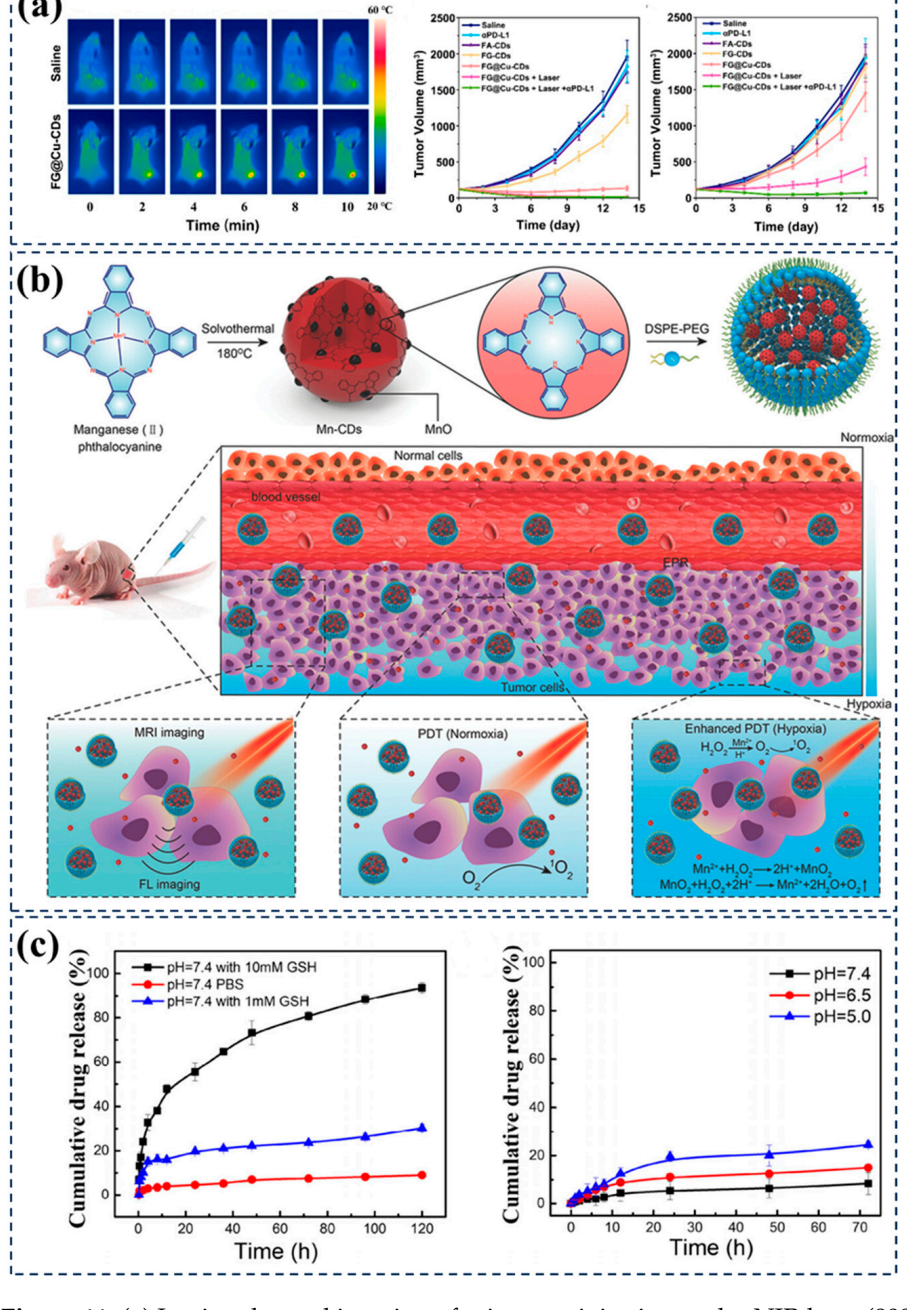
(**a**) In vivo thermal imaging of mice post injection under NIR laser (808 nm, 1.0 W cm^−2^). Primary tumor volume and distant tumor volume of the treated mice in different groups. Data presented as mean ± SD (*n* = 5). Reproduced with permission [122]. Copyright 2023, American Chemical Society. (**b**) Schematic illustration of the Mn-CD assembly as an acidic H_2_O_2_-driven oxygenerator to enhance the anticancer efficiency of PDT in a solid tumor. Reproduced with permission [80]. Copyright 2018, Wiley-VCH. (**c**) Controlled release profiles of DOX@HMSN-SS-C-dots-PLL (cit) at different concentrations of GSH (0, 1, and 10 mM) and the release behavior of DOX from DOX@HMSN-SS-C-dots-PLL (cit) at pH = 7.4, 6.5 and 5.0 (**c**). Reproduced with permission [126]. Copyright 2021, Springer Nature.

Wu et al. prepared r-CDs by mixing citric acid and urea in deionized water and utilizing microwave-assisted hydrothermal method. Further, the formation of f-CDAs was achieved through induced dehydration between particles [119]. These CDs exhibited significant PCE under laser irradiation at specific wavelengths, effectively elevating local temperatures to kill tumor cells. Jiang et al. synthesized near-infrared CDs with high PCE using ICG as the precursor [120]. The PCE of ICGCDs reached 23.9%, nearly 50% higher than the precursor ICG’s 16.3%. The enhanced optical properties make ICGCDs excel in applications such as biological imaging and PTT, especially as near-infrared imaging agents and photothermal therapeutic agents. Recently, Li et al. synthesized iron–copper-doped folic acid CDs (CFCFB) using folic acid and copper gluconate as precursors through multistep reactions [121]. CFCFB demonstrated powerful photothermal performance in the study, with a PCE of 55.8%. This characteristic renders CFCFB potentially valuable in PTT. In 2022, Bao et al. synthesized copper-doped CDs with dual responsiveness (FG@Cu-CDs) using folic acid (FA), gallic acid (GA), and copper sulfate (CuSO_4_) as precursors through a hydrothermal method [122]. The selection of biocompatible precursors such as folic acid and gallic acid for CDs, along with the use of copper sulfate as a source of copper, was based on their potential in anti-tumor drug research, particularly folate’s targeting ability to tumor cells. In animal model studies, the researchers evaluated the anti-tumor effects of FG@Cu-CDs by establishing tumor models in mice and intravenously injecting FG@Cu-CDs. Through near-infrared imaging technology, the researchers monitored the distribution of FG@Cu-CDs in mice and found significant accumulation at tumor sites with less distribution in other major organs, demonstrating good tumor targeting. As treatment progressed, the researchers observed a significant slowdown in tumor growth in the FG@Cu-CDs-treated group, especially in groups combining PTT and immune checkpoint inhibitors, with a significant reduction in tumor volume and even distant effects, indicating growth inhibition in distant tumors not directly treated (Figure 11a). Furthermore, histopathological analysis showed significant cell death and apoptosis features in tumor tissues treated with FG@Cu-CDs, while no significant damage was observed in normal tissues, indicating good safety and therapeutic efficacy of FG@Cu-CDs. This indicates that FG@Cu-CDs can convert light energy into heat energy under NIR irradiation, generating local high temperatures to directly ablate tumor tissues. Additionally, FG@Cu-CDs can generate highly toxic hydroxyl radicals (-OH) inside tumor cells through a Cu(I)-mediated Fenton-like reaction, inducing the apoptosis of tumor cells. The synergistic effect of PTT and chemodynamic therapy (CDT) effectively inhibits tumor growth.

Photodynamic therapy: PDT is a minimally invasive tumor treatment technique that combines photosensitizers with specific wavelength light sources. It selectively destroys tumor cells through photochemical reactions, offering advantages such as good tissue selectivity, low toxicity, and strong repeatability. It can also synergize with surgery, radiotherapy, chemotherapy, and other methods to improve efficacy, providing a safe and reliable new treatment option for cancer patients [123]. The basic principles of PDT involve three key elements: oxygen, photosensitizers, and laser. In this treatment process, when photosensitizers are introduced into the patient’s body and accumulate in the diseased tissue, laser irradiation of specific wavelengths triggers the production of reactive oxygen species by the photosensitizers. ROS can react with unsaturated lipids in the cell membrane, leading to the destruction of cell structure and function, effectively inhibiting and eliminating tumor cells while maximizing the protection of surrounding healthy tissues [36].

ZiXuan Li et al. prepared CDs with NIR emission characteristics and ROS production ability using dihydroxyphenyl e6 (Ce6) and polyethyleneimine as carbon sources via a solvothermal method, enabling NIR afterglow imaging and PDT therapy [124]. Jia et al. successfully synthesized Mn-CDs through multiple steps including solvothermal synthesis and self-assembly using manganese(II) phthalocyanine as a precursor [80]. Mn-CDs exhibited a strong fluorescence emission at 745 nm in the NIR region and efficient generation of singlet oxygen, enabling them to act as nano-photosensitizers to kill cancer cells under light irradiation (Figure 11b). The precursor manganese(II) phthalocyanine directly influenced the properties of the final Mn-CDs, imparting them with magnetism and enhancing their ability to catalyze the decomposition of hydrogen peroxide (H_2_O_2_) into oxygen, crucial for improving the hypoxic tumor microenvironment and enhancing the efficiency of PDT. Tian et al. successfully prepared nitrogen-doped CDs/mesoporous silica nanoparticles (NCDs/MSN) composite materials through a simple one-pot strategy [125]. Experimental results using electron spin resonance (ESR) spectroscopy and fluorescent probes such as DHR123 and DCFH-DA demonstrated that NCDs/MSN could effectively generate superoxide anions (O^2−^) and hydroxyl radicals (-OH) under 640 nm light irradiation. In vitro cytotoxicity experiments using Dihydroethidium (DHE) and hydroxyphenyl fluorescein (HPF) as probes showed that NCDs/MSN significantly increased the generation of reactive oxygen species inside cells under light conditions. Further evaluation of the anti-tumor effects in vivo in a BALB/c mouse model carrying 4T1 tumors confirmed the therapeutic efficacy of NCDs/MSN. The measurement of tumor volume and monitoring of mouse body weight revealed significant inhibition of tumor growth in the NCDs/MSN-treated group without apparent systemic toxicity. Additionally, histological analysis, including H&E staining and TUNEL staining, revealed a significant increase in cell apoptosis in the tumor tissue treated with NCDs/MSN, further confirming their effective PDT against hypoxic tumors in vivo.

### 3.4. Drug Delivery

Drug delivery technology delivers drugs precisely to specific parts of the body, enabling efficient treatment and reducing side effects. CDs, as an advanced drug carrier, exhibit tremendous potential in anticancer therapy due to their excellent physicochemical properties and biocompatibility [29]. The sp^2^ conjugated structure and surface functional groups of CDs not only facilitate the effective binding of drugs but also enhance their stability and targeting during treatment. Additionally, their fluorescent properties can be utilized to observe drug accumulation, aiding researchers in evaluating the therapeutic efficacy of drugs.

Chen et al. developed a novel nanocarrier incorporating NIR CDs [42], doxorubicin hydrochloride (DOX), hollow porous silica nanoparticles, and poly-l-lysine modified with citraconic anhydride (PLL), aiming to achieve high sensitivity to the tumor microenvironment [126]. Besides exhibiting NIR emission properties, the material was specifically designed to recognize and respond to specific conditions in the tumor microenvironment, such as acidic pH and higher concentrations of the reducing agent glutathione (GSH). The dual responsiveness to pH and GSH means that it can be activated in the acidic environment and high GSH concentration within tumor cells while remaining stable under normal physiological conditions (Figure 11c). This sensitivity and dual responsiveness enable it to trigger drug release in the environment surrounding tumor cells, thereby achieving targeted therapy for tumors while reducing the impact on normal tissues. Tan et al. prepared red-emitting CDs using electrochemical exfoliation of K_2_S_2_O_8_, which do not vary with excitation wavelength [127]. These CDs, while possessing good water solubility and biocompatibility, can label the cell membrane and cytoplasm of Hela cells, making them suitable for drug targeting delivery due to their chemically modifiable surface.

## 4. Conclusions and Future Outlook

CDs have garnered considerable attention due to their outstanding photostability, water solubility, and biocompatibility. Among them, R/NIR-CDs exhibit not only these advantages but also low intrinsic fluorescence, extremely low cytotoxicity, and excellent deep tissue penetration, which provide greater advantages in biological applications. This review discusses and analyzes the precursor materials used in the preparation of R/NIR-CDs and their impact on the emission wavelength of CDs. This article first introduces the common mechanisms for the generation of long-wavelength luminescence in CDs, with four widely accepted mechanisms: carbon core state, surface state, molecular state, and cross-linking enhanced emission effect. A systematic analysis is conducted on the specific effects of carbon sources and solvents on these mechanisms. Finally, we present the latest applications of R/NIR-CDs in bioluminescence imaging, biosensing, PTT, PDT, and drug targeting.

Although significant progress has been made in the synthesis, biomedical imaging, biosensing, and other applications of R/NIR-CDs, there are still several urgent issues to address in advancing their biomedical applications at present.

1. Current synthesis methods may perform well under small-scale laboratory conditions, but they may face challenges such as low yield, high cost, and difficulty in maintaining consistent product quality during large-scale production. Additionally, impurities generated during the synthesis process can affect the performance of CDs. Therefore, developing economically feasible and reproducible synthesis techniques to achieve large-scale production of high-quality R/NIR-CDs is a significant challenge.
2. Despite numerous studies analyzing the nucleation and growth mechanisms of R/NIR-CDs, the current understanding remains inadequate, limiting precise control over their structure and performance. Properties such as size, shape, defects, and functional groups significantly influence their optical properties and biocompatibility. Thus, in-depth research and understanding of these fundamental processes are crucial for optimizing synthesis conditions, enhancing carbon dot performance, and achieving customized synthesis.
3. Impurities introduced during the synthesis process, such as unreacted precursors, by-products, and other organic or inorganic impurities, may affect the optical properties and biocompatibility of R/NIR-CDs. Currently available purification methods may not completely remove these impurities, or the purification process may result in substantial material loss. This makes it difficult for researchers to analyze the impact of impurities on R/NIR-CDs. Developing new effective purification techniques is key to driving their application.
4. Inadequate understanding of the distribution, metabolism, and excretion processes of R/NIR-CDs in biological systems may affect their application in the biomedical field. Furthermore, detailed studies on long-term biocompatibility and potential toxicity issues are necessary to ensure the safety of R/NIR-CDs in clinical applications.
5. Despite the enormous potential of R/NIR-CDs in the biomedical field, their performance and effects in practical applications still need to be validated and optimized through extensive experiments and clinical trials. For example, research on the specific mechanisms of action of R/NIR-CDs in vivo, their interactions with biomolecules, and their effects in disease models are still lacking. Additionally, further exploration is needed to understand the applicability and limitations of R/NIR-CDs in different biological systems.

In addition to the aforementioned issues, the future development of R/NIR-CDs should focus on extending the emission wavelength of CDs to the NIR-II region to enhance the depth and resolution of bioimaging while reducing tissue absorption and scattering of light. Furthermore, enhancing the water solubility, stability, and biocompatibility of R/NIR-CDs is crucial for their application in the biomedical field. This includes enhancing their stability and biocompatibility in biological systems through surface modification and minimizing immune responses. Integrating other photoactive materials, such as aggregation-induced emission (AIE) agents and photothermal conversion agents, will bring new functionalities and applications to R/NIR-CDs, leading to the development of multifunctional nanocomposites with synergistic effects.

## Figures and Tables

**Table 1:** Summary of FL properties and luminescence mechanisms of R/NIR-CDs. The unlabeled data are measured in water by default.

The Main Mechanism	Carbon Source	Solvent	Ex/Em (nm)	QY (%)	Ref.
Carbon core state	3,4,9,10-perylenetetracarboxylic dianhydride, urea	DMF	720/745	3.30	[55]
	Manganese (II) phthalocyanine	alcohol	690/745	-_(in DMF)_	[80]
	o-Phenylenediamine, l-glutamic acid	H_2_SO_4_	620/715	36_(in water)_ and 43_(in ethanol)_	[58]
	o-Phenylenediamine, dopamine	HCl, water	540/710	26.28_(in ethanol)_	[65]
	o-Phenylenediamine, AlCl_3_·6H_2_O	-	532/700	57	[53]
	glutathione, urea	formamide	420/696	14.3	[81]
	taxus leaves	acetone	413/673	45_(in acetone)_	[4]
	citric acid,	DMF, NH_3_H_2_O	580/667	60.73	[61]
	citric acid, urea, sodium carbonate	formic acid	535/660	14	[82]
	citric acid	DMF, formamide	560/655	26.8	[83]
	citric acid, o-phenylenediamine	acid solvent	600/635	24.99	[84]
	o-Phenylenediamine	HNO_3_	540/630	10.83 _(in water)_ and 31.54_(in ethanol)_	[67]
	citric acid, thiourea	DMF, NaOH, HCl	550/610	24	[85]
	p-Phenylenediamine	DMF	520/608	40	[52]
	1,8-diaminonaphthalene, critic acid	DMF	514/607	30.68_(after surface modification)_	[86]
	1,1′-binaphthyl-2,2′-diamine, citric acid	toluene	540/603	13.3	[87]
Surface state	o-Phenylenediamine, dopamine hydrochloride, FeCl_3_·6H_2_O	water	830/1000	1.27_(in acid solvent)_	[64]
	1,5-diaminonaphthalene, ammonium citrate	DMF	705/790	1.8	[88]
	citric acid, urea	DMF	732/760	26_(in DMSO)_	[7]
	citric acid, urea	DMSO	655/720	0.20	[66]
	o-Phenylenediamine, tert-butyl hydroperoxide	HCl, water	610/653	8.1	[89]
	polyethyleneimine, reduced glutathione	water	420/650	-	[90]
	p-Phenylenediamine	Dimethylacetamide, NaOH	540/640	29.1	[91]
	2,7-dihydroxynaphthalene, citric acid, L-methionine	DMF, water	490/640	27.56	[25]
	citric acid	formamide	540/640	16.2_(in water)_ and 22.9_(in methanol)_	[92]
	citric acid, polyethyleneimine, chlorin e6	formamide	550/640	-	[93]
	o-Phenylenediamine	H_2_SO_4_	612/629	17	[63]
	citric acid, urea	DMF	561/624	6_(in water)_	[7]
	1,3,6-trinitropyrene	DMF	550/620	27.40	[68]
	urea, neutral red	water	470/620	15.96_(in water)_ and 61.11_(in acetone)_	[94]
	citric acid, urea	DMF	550/620	9.75	[95]
	p-Phenylenediamine, ammonium thiocyanate	water	598/618	8.7_(in water)_ and 17.58_(in ethanol)_	[96]
	1,2,4,5-Benzenetetracarboxylic acid, 2,7-diaminofluorene	ethanol	500/605	10	[97]
	citric acid, urea	DMF	550/600	12.90	[6]
	citric acid, thiourea,	acetone	560/594	22	[9]
	1,2,4-triaminobenzene dihydrochloride, urea	water	500/585	55	[69]
Molecular state	Cy-COOH, PEG600	methanol	620/710	18.8	[98]
	citric acid, ICG, PEG1000	water	620/697	-	[74]
	tetraphenylporphyrin	H_2_SO_4_, HCl	435/692	23.8	[99]
	azure A chloride, copper gluconate	water	658/683	5	[73]
	o-Phenylenediamine, catechol, AlCl_3·_6H_2_O	-	520/650	2.65_(in ethanol)_	[72]
	o-Phenylenediamine, acetic acid	ethanol, H_2_SO_4_	600/620	16.47	[78]
	neutral red	ethylene glycol	535/620	7	[100]
	sodium sulfate, o-Phenylenediamine	water	610/620	1.9_(in acid solvent)_	[101]
	p-Phenylenediamine	toluene	510/608	31.4_(in ethanol)_	[60]
	citric acid, m-aminophenol	water	572/600	40	[71]

## Data Availability

No new data were created or analyzed in this study.
